# Effects of astaxanthin on blood coagulation, fibrinolysis and platelet aggregation in hyperlipidemic rats

**DOI:** 10.1080/13880209.2016.1261905

**Published:** 2016-12-12

**Authors:** Zu-Yue Deng, Wei-Guang Shan, Shen-Feng Wang, Meng-Mei Hu, Yan Chen

**Affiliations:** a College of Pharmaceutical Science, Zhejiang University of Technology, Hangzhou, China;; b Zhejiang Institute for Food and Drug Control, Hangzhou, China

**Keywords:** Carotenoid, antioxidant activity, t-PA/PAI-1 balance, TXA2/PGI2 balance, NO/ROS balance

## Abstract

**Context:** Astaxanthin (ASTX) is a xanthophyll carotenoid that reduces hemostasis in hyperlipidemic organisms. Its antihemostatic mechanisms remain unclear.

**Objective:** The effects of ASTX on coagulation, the fibrinolytic system and platelet aggregation were investigated in hyperlipidemic rats.

**Materials and methods:** Different doses of ASTX (5, 10 and 30 mg/kg/day, p.o.) were administered for four weeks to high-fat diet-induced hyperlipidemic rats. Serum lipid and lipoprotein levels were measured with an automatic biochemical analyzer. The prothrombin time (PT), activated partial thromboplastin time (APTT) and maximum platelet aggregation rate (MAR) were determined by a coagulation analyzer. The activities of the tissue-type plasminogen activator (t-PA), type-1 plasminogen activator inhibitor (PAI-1) and endothelial nitric oxide synthase (eNOS), as well as the levels of thromboxane B(2) [TXB(2)], 6-keto prostaglandin F(1α) [6-keto-PGF(1α)] and platelet granule membrane protein (GMP-140), were measured with enzyme-linked immunosorbent assay kits. Gene and protein expression levels were analyzed by reverse transcriptase polymerase chain reaction and Western blot, respectively.

**Results:** ASTX (30 mg/kg) treatment in hyperlipidemic rats reduced serum TG (0.58 ± 0.14 versus 1.12 ± 0.24 mmol/L), serum TC (1.77 ± 0.22 versus 2.24 ± 0.21 mmol/L), serum LDL-C (1.13 ± 0.32 versus 2.04 ± 0.48 mmol/L), serum MDA (69%), plasma MAR (55%), serum TXB2/6-keto-PGF1α (34%) and serum GMP-140 levels (25%), plasma PAI-1 activity (48%) and downregulated the mRNA (33%) and protein (23%) expression of aorta eNOS, the mRNA (79%) and protein (72%) expression levels of aorta PAI-1. However, ASTX (30 mg/kg/d) treatment increased serum SOD activity (2.1 fold), serum GPx activity (1.8 fold), plasma PT (1.3 fold), plasma APTT (1.7 fold), serum NO (1.4-fold), serum 6-keto-PGF1α (1.3 fold).

**Conclusions:** ASTX reduced blood coagulation and platelet aggregation and promoted fibrinolytic activity in hyperlipidemic rats. These activities were closely correlated with ASTX, maintaining the balance of t-PA/PAI-1, NO/ROS and TXA2/PGI2 *in vivo*.

## Introduction

The excessive intake of high-lipid food and the dramatic changes in lifestyle easily cause hyperlipidemia. The hyperlipidemia is clinically considered a major risk factor of cardiovascular diseases, which is the second leading cause of death worldwide. A previous study (Bai et al. 2006) revealed that patients with cardiovascular disease usually have lipid disorders and are in a pro-coagulant state. Therefore, anticoagulant treatment plays an important role in reducing the risk of cardiovascular diseases.

Over the past few decades, numerous anticoagulant drugs have been applied for clinical treatment. The advantageous low toxicity of natural agents has attracted attention in anticoagulant research.

Astaxanthin (3,3-dihydroxy-β,β-carotene-4,4-dione; ASTX) belongs to the xanthophyll subclass of carotenoids (Santos et al. 2012). A green alga, *Haematococcus pluvialis* contains the highest level of ASTX (up to 4%/g, dry weight), and it is a main source of natural ASTX (Yang et al. 2011). Given its physicochemical properties and high-added value, ASTX is widely used in feed additives, cosmetics and pharmaceuticals (Santos et al. 2012). Apart from its stronger antioxidation ability, ASTX shows other effects, such as immune response enhancement, cancer risk reduction and neuroprotection. The effects of ASTX in the haemostatic system have been reported. Sasaki et al. (2011) studied the antithrombotic and antihypertensive effects of ASTX. Lauver et al. (2008) also found that the disodium disuccinate derivative of ASTX significantly reduces secondary thrombosis while maintaining normal haemostasis. However, Serebruany et al. (2010) reported that ASTX *in vitro* does not affect the platelet, coagulation or fibrinolytic indices in either aspirin-naive or aspirin-treated subjects. The different results are mainly caused by the incomplete understanding of the mechanisms behind the antihemostatic effects of ASTX. This study was designed to systematically investigate the effects of ASTX on various hemostatic indices and the mechanisms underlying the antihemostatic effects of ASTX in hyperlipidemic rats. A hyperlipidemia rat model fed with a high-fat diet was used, and the hemostatic parameters were investigated after ASTX treatment of hyperlipidemic rats. The effects and mechanisms of ASTX on coagulation, fibrinolytic activity, and platelet aggregation were revealed. We found that ASTX demonstrated anticoagulation properties, inhibited platelet aggregation, and improved fibrinolytic activity. The underlying mechanisms were related to the lowered lipid levels, production of antioxidants and protection of endothelial cells. ASTX was also found to be involved in the balance of t-PA/PAI-1, NO/ROS and TXA2/PGI2.

## Materials and methods

### Chemicals

ASTX was purchased from ZheJiang Conba Pharmaceutical Co., Ltd. (Batch number: 20140128; purity: ≥92%; Hangzhou, China). All other chemicals were purchased from Sinopharm Chemical Reagent Co., Ltd. (Shanghai, China) unless otherwise specified. All commercially available chemicals used in the measurements were of the highest purity.

### Dose selection, chemical preparation and duration of supplementation

ASTX was stored in dark and anoxic conditions at a low temperature (−20 °C) before preparing the oral oil solution. The ASTX dosage used was based on the literature (Tripathi & Jena 2010; Choi et al. 2011). ASTX was diluted in olive oil (Meidikang, China) at concentrations of 5, 10 and 30 mg/mL. ASTX was freshly prepared and diluted in olive oil immediately before use. The selected duration of ASTX supplementation was based on the hyperlipidemic rat model because lipid disorders are significant during 4–8 weeks of treatment.

### Animals

A total of 72 male Sprague–Dawley rats (Certificate No. SCXK (hu) 2012-0002) were purchased from Shanghai SLRC Laboratory Animal Company Limited (Shanghai, China). The experiments were approved by the laboratory animal ethics committee of the Zhejiang Provincial Research Institute for Food and Drug Control and followed the NIH guidelines for the care and use of laboratory animals (NIH Publication No. 80-23, revised 1978). The rats were individually housed in cages at 23 °C ± 3 °C on a 12 h light:dark cycle, with free access to regular rodent chow and water. The weights of the rats at the beginning of the study ranged from 172 to 205 g. The animals were randomly divided into six groups after a week of acclimatization, with 12 rats in each group. The groups were as follows: a normal control group (NC), an ASTX group (10 mg ASTX/kg/d in olive oil), a high-fat control group (HC), a low-dose HC + ASTX group (high-fat diet + 5 mg ASTX/kg/d in olive oil), a middle-dose HC + ASTX group (high-fat diet + 10 mg ASTX/kg/d in olive oil; the dosage is based on human common use dose) and a high-dose HC + ASTX group (high-fat diet + 30 mg ASTX/kg/d in olive oil). Rats from the NC and ASTX groups were fed standard diets, whereas the other groups were fed high-fat diets (standard diet of 78.8% (w/w) supplemented with pig oil (10%, w/w), powdered egg yolk (10%, w/w), cholesterol (2%, w/w) and bile salt (0.2%, w/w); the composition of high-fat diets: 26.2% protein, 26.3% carbohydrate and 34.9% fat, with a calorie composition of 20 kcal%, 20 kcal% and 60 kcal%, respectively) for eight weeks. ASTX was intragastrically administered in the ASTX-treated groups every afternoon for the final four weeks after being fed with the high-fat diet for the first four weeks. Rats in the NC and HC groups were orally administered with distilled water of the same volume. The body weights (BWs) of the rats were measured weekly for 8 weeks, and the general condition of the animals was observed daily.

### Collection of blood samples and thoracic aorta tissues

The rats were fasted for 12 h and sacrificed after four weeks of treatment. Blood samples were collected from the abdominal aorta. Serum was separated by centrifugation at 4 °C and stored at −20 °C for biochemical analysis. Plasma was separated for the determination of haematological parameters. Each plasma sample was divided into half and prepared according to the following process. Half of each blood sample was treated with 109 mmol/L sodium citrate solution at a ratio of 1:9 to prevent coagulation, followed by centrifugation at 700 rpm for 15 min to separate the platelet-rich plasma (PRP). The remaining sample was centrifuged at 2500 rpm for 15 min to separate the platelet-poor plasma (PPP). The PRP and PPP were used to determine platelet aggregation. The second half of each blood sample was treated with 109 mmol/L sodium citrate solution at a ratio of 1:9 to prevent coagulation and then centrifuged at 2500 rpm for 15 min. This prepared plasma sample was used for the prothrombin time (PT) and APTT tests. All haematological parameters were measured within 4 h of sample preparation. The aortic arches from individual rats were subjected to gene expression and Western blot analyses.

### Serum biochemical analysis

The serum TC, triglyceride (TG), low-density-lipoprotein cholesterol (LDL-C), high-density-lipoprotein cholesterol (HDL-C), lipoprotein (a) (LP(a)), fibrinogen (FG), D-dimer (D-D), apolipoprotein E (apoE), apolipoprotein AI (apoAI) and apolipoprotein B100 (apoB100) levels were measured with a Hitachi 7020 automatic biochemical analyzer (Hitachi, Japan) and kits from Shanghai Shenergy-Diasys Diagnostics Technology Co., Ltd (Shanghai, China).

### Determination of the serum SOD, GPx, and eNOS activity and the MDA, NO, GMP140, TXB2 and 6-keto-PGF1α levels

The serum GMP140, TXB2, and 6-keto prostaglandin F (1α) (6-keto-PGF1α) levels were measured with enzyme-linked immunosorbent assay (ELISA) kits (Soochow University, China). Serum superoxide dismutase (SOD) activity in the serum was determined by monitoring the inhibition of autoxidation of hydroxylamine (Zhu et al. 2011) using a SOD-detection assay kit (Jiancheng Bioengineering, Nanjing, China) according to the manufacturer’s instructions. One unit of SOD activity was determined as the amount of enzyme that inhibited the autoxidation of hydroxylamine by 50%. SOD activity was expressed as U/mL. Glutathione peroxidase (GPx) activity in the serum was determined spectrophotometrically by measuring the content of reduced glutathione (GSH) (Liu et al. 2013) using a GPx-detection assay kit (Jiancheng Bioengineering) according to the manufacturer’s instructions. One unit was determined as the amount of enzyme that catalyzed the reduction in GSH level in the reaction system by 1 μmol/L. GPx activity was expressed as U/mL. The levels of malondialdehyde (MDA) in serum were determined spectrophotometrically by measuring the content of thiobarbituric acid reactive substance (TBARS) (Ohkawa et al. 1979) using an MDA-detection assay kit (Jiancheng Bioengineering, Nanjing, China) according to the manufacturer’s instructions. The levels of MDA were expressed as nmol/mL. Serum total NOS (tNOS) and inducible NOS (iNOS) activity were assayed using commercial kits. The tNOS activity minus the iNOS activity gave us the constitutive NOS (cNOS) activity. In rat serum, the main cNOS is endothelium-derived NOS (eNOS) (Förstermann 2010). The nitric oxide (NO) levels were measured using a NO-detection assay kit (Jiancheng Bioengineering, Nanjing, China) according to the manufacturer’s instructions. This assay kit contained a microtiter plate, nitrate reductase, nitrate reductase storage buffer, NADH, nitrate standard, nitrite standard, reaction buffer concentrate, Griess reagent I and Griess reagent II.

### Determination of plasma PT and APTT

The plasma PT and APTT levels were measured using an LG-PABER-1Ch Coagulation Analyzer (Steellex Scientific Instrument Company, Beijing, China) with a clot formation kit, according to the manufacturer’s protocol. The experimental conditions of the tests were maintained at 25 °C with 60% humidity.

### Determination of the plasma tissue plasminogen activator and plasminogen activator inhibitor-1 activities

Tissue plasminogen activator (tPA) and plasminogen activator inhibitor-1 (PAI-1) activities were measured with ELISA kits (Hefei Bomei Biotechnology Co. Ltd., Hefei, China) according to the manufacturer’s instructions. Results are expressed in units per milliliter (IU/mL or AU/mL).

### Determination of platelet aggregation

Platelet formation (cell density of 400–600 × 10^9^ cells/L) was induced by 5 μmol/L adenosine diphosphate. Platelet aggregation was observed in terms of the speed of change in light transmission over time. The maximum extent of platelet aggregation (5 min) was expressed as the platelet maximal aggregation rate (MAR). This parameter was recorded by a LG-PABER-1CH Coagulation Analyzer (Steellex Scientific Instrument Company, Beijing, China). The experimental conditions of the tests were maintained at 20 °C with 60% humidity.

### RNA extraction and quantitative real-time polymerase chain reaction (PCR)

The relative eNOS, t-PA and PAI-1 mRNA levels were determined by real-time PCR and compared with those of the control gene, glyceraldehyde-3-phosphate dehydrogenase (GAPDH). Total RNA was extracted from the frozen thoracic aorta from the different rat groups using TRizol reagent (Takara, Dalian, China). cDNA synthesis was performed using MuLVReverse Transcriptase (Takara, Dalian, China). Real-time PCR was performed with a SYBR Green PCR master mix kit (Roche, USA). Primer sequences were designed according to the GenBank database using Beacon Designer software (PREMIER Biosoft, Roche). Primer sequences are listed in Table 1. The PCR reactions were performed at 95 °C for 30 s, followed by 40 cycles of 95 °C for 10 s, 55 °C for 10 s and 72 °C for 30 s. PCR reactions were performed in triplicate for each sample-primer set, and the mean of the three experiments was used as the relative quantification value. Melting curves were determined at the end of each PCR cycle to check product purity. The data were analyzed using Roche-gene software (Roche, USA). The relative expression levels of each gene were determined by the 2^−ΔΔCt^ method.

**Table 1.** Primers used for real-time PCR analysis.

Gene	Forward primer (5′–3′)	Reverse primer (5′–3′)	Length (bp)
eNOS	GACCAGCACCAGACCACA	TGCACTGAGGGTATCGTAGGT	110
t-PA	GCTGAATGCATCAACTGGAA	CGTCTCGGTCTGGGTTTCT	123
PAI-1	AGGAGGAACGCTGCACAC	AGTGAGGGCTGAAGACATCTG	76
GAPDH	CTACCCACGGCAAGTTCAAT	ATTTGATGTTAGCGGGATCG	83

### Western blot analysis

Thoracic aorta samples (0.5 g) were homogenized in 1 mL of RIPA lysis buffer (Beyotime Institute of Biotechnology, China) containing the protease inhibitor cocktail set III (Beyotime). Tissue lysates were incubated on ice for 30 min before the lysates were centrifuged at 12,000 ×*g* for 20 min at 4 °C. The supernatant was collected, and the protein concentrations were measured using a Coomassie Blue Staining Kit (Beyotime), with bovine serum albumin as the standard. Samples containing 50 μg of protein were resolved by 10% sodium dodecyl sulfate PAGE, electrotransferred onto a nitrocellulose membrane and incubated with antibodies against eNOS, t-PA, PAI-1 (Bioworld, USA) and GAPDH (Beyotime), according to the manufacturer’s instructions. Protein bands were developed with a horseradish peroxidase system (Beyotime). Protein bands were visualized by enhanced chemiluminescence with a BeyoECL Plus kit (Beyotime). The resulting images were resolved with Kodak X-Omat BT film (5 × 7IN; Beyotime). Band intensity was quantified by Gel-Pro Analyzer software (Liu Yi, Beijing). GAPDH was used as the loading control.

### Statistical analysis

Data were expressed as the mean ± standard deviation (SEM). The values were calculated from the specified number of determinations with SPSS statistical software package (version 17.0; SPSS, Chicago, IL). Significant differences between the experimental and control groups were assessed by Student’s *t*-test (data analysis of two experimental groups) and one-way ANOVA (data analysis of multi-experimental groups). Values with *p* < 0.05 were considered statistically significant.

## Results

### Effect of ASTX on the BW and biochemical parameters in hyperlipidemic rats

No significant differences in the BW and serum biochemical parameters were observed between the ASTX and NC groups, thereby suggesting that treatment with ASTX alone did not affect the normal BW and serum biochemical parameters.

However, levels of TG (1.12 ± 0.24 mmol/L vs. 0.49 ± 0.08 mmol/L), TC (2.24 ± 0.21 mmol/L vs. 1.43 ± 0.12 mmol/L), LDL-C (2.04 ± 0.48 mmol/L vs. 1.11 ± 0.22 mmol/L), apoB100 (1.42 ± 0.12 g/L vs. 1.08 ± 0.12 g/L), apoE (3.41 ± 0.78 mg/L vs. 3.09 ± 1.03 mg/L), LP(a) (43.28 ± 2.67 mg/L vs. 13.08 ± 4.72 mg/L), D-D (2.64 ± 0.44 mg/L vs. 1.85 ± 0.24 mg/L), and FG (2.32 ± 0.35 mg/L vs. 2.02 ± 0.44 mg/L) in the HC group were elevated compared with those in the NC group (*p* < 0.01 or *p* < 0.05 across all comparisons). By contrast, the levels of HDL-C (1.35 ± 0.16 mmol/L vs. 1.69 ± 0.18 mmol/L) decreased (*p* < 0.05) in the HC group, the Atherogenic Index was 0.65, while those of apoAI (0.98 ± 0.07 g/L vs. 1.02 ± 0.09 g/L) were not significantly different (*p* > 0.05). These results indicated that an experimental rat model of hyperlipidemia was successfully established.

**Figure 1.**
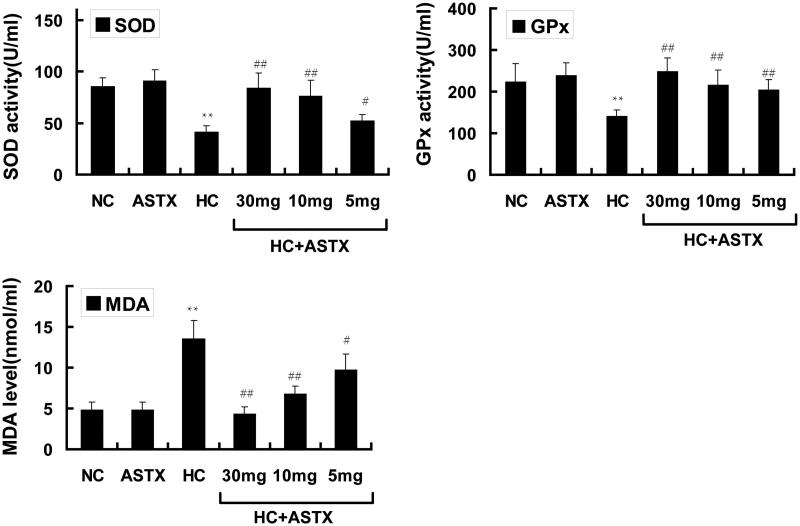
Antioxidative effects of ASTX on lipid peroxidation and the activity of SOD and GPx. Experimental rats were fed with a high-fat diet before treatment with solvent or ASTX. The amount of SOD (A), GPx (B) activities and MDA levels or lipid peroxidation (C) in the blood samples were determined. Data are expressed as the amount of SOD or MDA, mean ± SEM; *n* = 10; **p < 0.01, compared with rats fed with a normal diet; #*p* < 0.05, ##*p* < 0.01, compared with rats fed with a high-fat diet.

However, compared with the HC group, ASTX caused a modest decrease in BW in the HC + ASTX groups. Treatment with 30, 10 and 5 mg/kg ASTX reduced the levels of TG (0.58 ± 0.14, 0.62 ± 0.12 and 0.71 ± 0.18 mmol/L vs 1.12 ± 0.24 mmol/L, respectively), TC (1.77 ± 0.22, 1.87 ± 0.22 and 1.83 ± 0.19 mmol/L vs 2.24 ± 0.21 mmol/L, respectively), LDL-C (1.13 ± 0.32, 1.23 ± 0.28 and 1.35 ± 0.22 mmol/L vs 2.04 ± 0.48 mmol/L, respectively), LP (a) (27.29 ± 4.27, 33.93 ± 7.17 and 37.96 ± 2.98 mg/L vs 43.28 ± 2.67 mg/L, respectively), and D-D (1.77 ± 0.25, 1.96 ± 0.23 and 2.08 ± 0.25 mg/L vs 2.64 ± 0.44 mg/L, respectively) (*p* < 0.01 or *p* < 0.05 across all comparisons). However, the same treatment mildly reduced the apoB100 (1.17 ± 0.11, 1.22 ± 0.13 and 1.31 ± 0.12 g/L vs 1.42 ± 0.12 g/L, respectively), apoE (3.05 ± 0.64, 3.21 ± 0.81 and 3.28 ± 0.32 mg/L vs 3.41 ± 0.78 mg/L, respectively) (Figure 1; 96 ± 0.32, 2.06 ± 0.46 and 2.16 ± 0.38 mg/L vs 2.32 ± 0.35 mg/L, respectively) levels (*p* < 0.05). By contrast, the levels of HDL-C (1.96 ± 0.15, 1.86 ± 0.15 and 1.68 ± 0.16 mmol/L vs 1.35 ± 0.16 mmol/L, respectively) increased (*p* < 0.01 or *p* < 0.05). In addition, the apoAI levels (1.14 ± 0.06, 1.04 ± 0.05 and 1.01 ± 0.07 g/L vs 0.98 ± 0.07 g/L, respectively) increased modestly in the HC + ASTX groups (*p* > 0.05) (Table 2).

**Table 2.** Biochemical parameters in various groups.

Groups	NC	ASTX	HC	H-ASTX 30mg	HC + ASTX M-ASTX 10 mg	L-ASTX 5 mg
TG (mmol/L)	0.49 ± 0.08	0.44 ± 0.08	1.12 ± 0.24^b^	0.58 ± 0.14^d^	0.62 ± 0.12^d^	0.71 ± 0.18^c^
TC (mmol/L)	1.43 ± 0.12	1.52 ± 0.12	2.24 ± 0.2l^b^	1.77 ± 0.22^d^	1.87 ± 0.22^c^	1.83 ± 0.19^c^
HDL-C (mmol/L)	1.69 ± 0.18	1.74 ± 0.15	1.35 ± 0.16^a^	1.96 ± 0.15^d^	1.86 ± 0.15^d^	1.68 ± 0.16^c^
LDL-C (mmo1/L)	1.11 ± 0.22	1.03 ± 0.14	2.04 ± 0.48^b^	1.13 ± 0.32^d^	1.23 ± 0.28^c^	1.35 ± 0.22^c^
ApoAI (g/L)	1.02 ± 0.09	1.03 ± 0.08	0.98 ± 0.07	1.14 ± 0.06	1.04 ± 0.05	1.01 ± 0.07
ApoB l00 (g/L)	1.08 ± 0.12	1.07 ± 0.09	1.42 ± 0.12^d^	1.17 ± 0.11^c^	1.22 ± 0.13	1.31 ± 0.12
ApoE (mg/L)	3.09 ± 1.03	3.11 ± 0.76	3.41 ± 0.78^c^	3.05 ± 0.64^c^	3.21 ± 0.81	3.28 ± 0.32
Lp (a) (mg/L)	13.08 ± 4.72	15.88 ± 6.00	43.28 ± 2.67^d^	27.29.±4.27^d^	33.93.±7.17^c^	37.96 ± 2.98
D-D (mg/L)	1.85 ± 0.24	1.76 ± 0.28	2.64 ± 0.44^d^	1.77 ± 0.25^d^	1.96 ± 0.23^d^	2.08 ± 0.25^c^
FIG (mg/L)	2.02 ± 0.44	1.97 ± 0.42	2.32 ± 0.35^a^	1.96 ± 0.32^c^	2.06 ± 0.46	2.16 ± 0.38

TG: triglyceride; TC: total cholesterol; HDL-C: High-density lipoprotein–cholesterol; LDL-C: low-density lipoprotein–cholesterol; ApoAI: apolipoprotein AI; ApoB100: apolipoprotein B100; ApoE: apolipoprotein E; Lp (a): lipoprotein (a); D-D: D-dimer; FIG: fibrinogen; NC: control; HC: high fat control group; ASTX: astaxanthin-treated. All values are mean ± SEM; *n* = 12 rats in all groups.

a *p* < 0.05.

b *p* < 0.01 versus NC group.

c *p* < 0.05.

d *p* < 0.01 versus HC group.

### Effects of ASTX on lipid peroxidation and antioxidative enzyme activity in rats

Different oxidization indices were determined to study the effects of ASTX on antioxidative activity in normal rats. Compared with the NC group, treatment with ASTX alone did not significantly influence SOD and GPx activities and did not decrease the MDA content. The HC group demonstrated a 2.8-fold elevation in the MDA content (*p* < 0.01; Figure 1(C)), but exhibited decreased SOD and GPx activities (52% and 37%, respectively; *p* < 0.01 or *p* < 0.05) in serum. Compared with the HC group, treatment with 30, 10 and 5 mg/kg ASTX significantly reduced the MDA content (69%, 50% and 38%; *p* < 0.01 or *p* < 0.05; Figure 1(C)) and increased the activities of SOD (2.1-, 1.9- and 1.3-fold; *p* < 0.01 or *p* < 0.05; Figure 1(A)) and GPx (1.8-, 1.6- and 1.5-fold, *p* < 0.01; Figure 1(B)).

### Effect of ASTX on the NO levels and eNOS activity in serum and eNOS expression in the aorta

**Figure 2.**
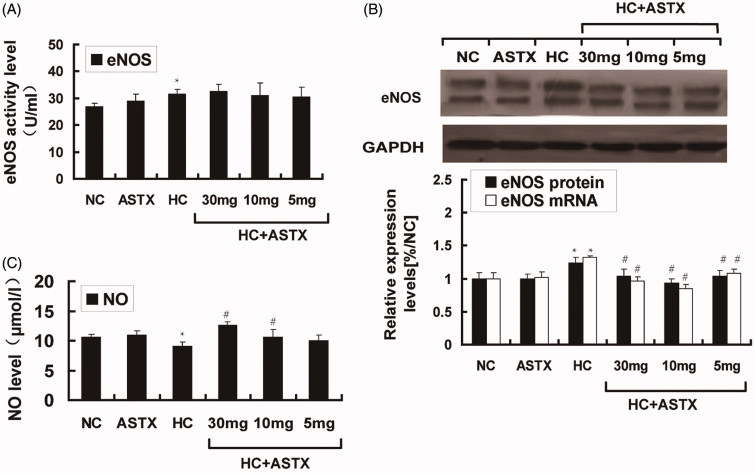
Effect of ASTX on NO levels, eNOS activity in serum and eNOS expression in the aortic arch. Hyperlipidemic rats were treated with solvent or ASTX, and the blood samples and aortic arches were analyzed. (A) Total RNA and eNOS mRNA expression levels in experimental rats fed with a high-fat diet were determined by qRT-PCR. GAPDH was used as the standard housekeeping gene. (B) Western blots show the eNOS protein in cell lysates of rats under a high-fat diet. Equal amounts of cellular protein extracts were loaded on 10% SDS-PAGE gels. A double band of eNOS protein (w136 kDa) was detected at different concentrations in all tested samples. GAPDH (w37 kDa) was used to control equal loading. eNOS activity (C) and NO levels (D) were analyzed with commercial kits. Data are expressed as mean ± SEM of blood samples (*n* = 10) and aortic arch samples (*n* = 3); *p < 0.05, compared with rats fed with a normal diet; #*p* < 0.05, ##*p* < 0.01, compared with rats fed with a high-fat diet.

Hyperlipidemia induces vascular endothelial dysfunction, and NO is closely related to endothelial function. We analyzed the changes in NO and eNOS in hyperlipidemic rats. No significant differences in the NO levels, eNOS activity and eNOS expression were observed between the ASTX and NC groups. Compared with the NC group, hyperlipidemic rats exhibited decreased NO levels (24%, *p* < 0.05; Figure 2(C)) and increased eNOS activity (1.2-fold, *p* < 0.05; Figure 2(A)) in the serum. The mRNA (1.3-fold, *p* < 0.05) and protein (1.4-fold, *p* < 0.05) expression levels of eNOS were altered in the aorta (Figure 2(B)). Compared with the HC group, ASTX was beneficial to the NO levels (1.4- and 1.2-fold; *p* < 0.05) only when administered at doses of 30 and 10 mg/kg (*p* < 0.05; Figure 2(C)). Treatment with 30, 10 and 5 mg/kg ASTX decreased the mRNA (33, 42 and 22%; *p* < 0.05) and protein (23, 32 and 21%; *p* < 0.05) expression of eNOS (Figure 2(B)) in the ASTX-treated groups. However, no significant differences were found in eNOS activity (Figure 2(A)) among the ASTX-treated groups.

### Effects of ASTX on the coagulation parameters PT and APTT in hyperlipidemic rats

**Figure 3.**
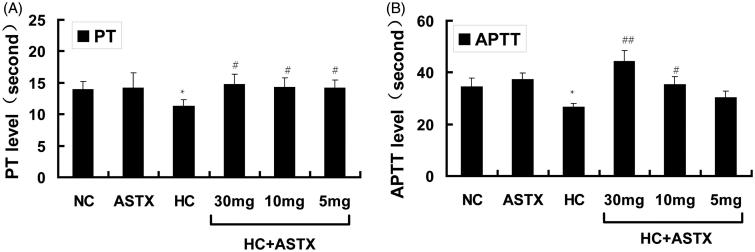
Anticoagulation effects of ASTX in hyperlipidemic rats. Experimental rats were fed with a high-fat diet and treated with solvent or ASTX. The coagulation index (PT and APTT) in the blood samples was determined with a coagulation analyzer. (A) PT levels and (B) APTT levels. Data are expressed as mean ± SEM (*n* = 12); *p < 0.05, compared with rats fed with a normal diet; #*p* < 0.05, ##*p* < 0.01, compared with rats fed with a high-fat diet.

PT is a biomarker of the intrinsic coagulation path, whereas APTT is a biomarker of the extrinsic coagulation path. The PT and APTT levels were determined to study the anticoagulation effects of ASTX on hyperlipidemic rats. Compared with normal rats, no differences in the PT and APTT levels were observed in the ASTX group. However, PT and APTT were reduced in hyperlipidemic rats (20 and 23%, *p* < 0.05; Figure 3(A,B)). Compared with the HC group, treatment with 30, 10 and 5 mg/kg ASTX significantly increased PT (1.3-, 1.3- and 1.3-fold; *p* < 0.05; Figure 3(A)). ASTX was beneficial to the APTT level in the HC + ASTX groups only when administered at doses of 30 and 10 mg/kg (1.7- and 1.3-fold, *p* < 0.01 or *p* < 0.05; Figure 3(B)).

### Effects of ASTX on the fibrinolytic parameters(tPA and PAI-1) of hyperlipidemic rats

**Figure 4.**
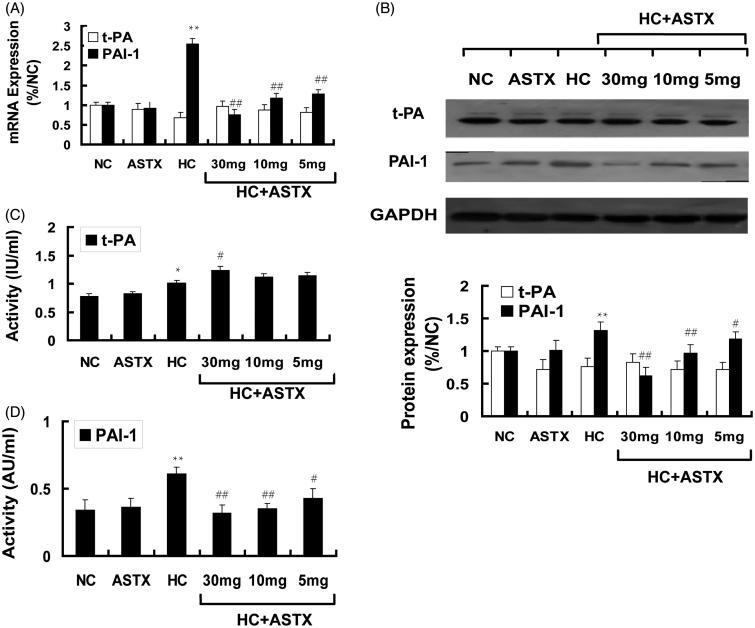
Effects of ASTX on the fibrinolytic parameters of hyperlipidemic rats. Hyperlipidemic rats were treated with solvent or ASTX. Blood samples and aortic arches were analyzed. (A) Total RNA from experimental rats fed with a high fat diet; tPA and PAI-1 mRNA expression levels were determined by qRT-PCR. The housekeeping gene GAPDH was used as the reference standard. (B) Western blots showing tPA and PAI-1 proteins in the cell lysates of rats fed with a high-fat diet. Equal amounts of cellular protein extracts were loaded on a 10% SDS-PAGE gel. A single band of tPA and PAI-1 protein (w65 and 110 kDa, respectively) was detected with various abundance levels in all the tested samples. GAPDH (w37 kDa) was used to control equal loading. (C) tPA activity was determined by ELISA (IU/mL). (D) PAI-1 activity was determined by ELISA (AU/mL). Data are expressed as mean ± SEM of blood samples (*n* = 10) and aortic arch samples (*n* = 3); *p < 0.05, **p < 0.01, compared with rats fed with a normal diet; #*p* < 0.05, ##*p* < 0.01, compared with rats fed with a high-fat diet.

The activities and relative levels of the t-PA and PAI-1 gene transcripts and their protein expression in the aorta were determined by qRT-PCR and Western blot analysis to explore the effects of ASTX treatment in hyperlipidemic rats. No significant differences were observed in the activity, gene expression and protein expression of t-PA and PAI-1 between the ASTX and NC groups. Similarly, no significant differences were observed for these parameters between the ASTX and NC groups. Compared with the NC group, hyperlipidemic rats demonstrated increased t-PA activity (1.2-fold, *p* < 0.05; Figure 4(C)) and PAI-1 activity (1.8-fold, *p* < 0.01; Figure 4(D)) in the serum, and mildly downregulated mRNA and protein expression of t-PA (Figure 2(A,B)). By contrast, the mRNA and protein expression levels of PAI-1 were significantly upregulated in the aorta (3.5- and 2.3-fold; *p* < 0.01; Figure 2(A,B)). Compared with the HC group, treatment with 30, 10 and 5 mg/kg ASTX significantly decreased PAI-1 activity (48%, 43% and 30%; *p* < 0.05; Figure 4(D)) and significantly downregulated the mRNA (79%, 68% and 64%; *p* < 0.01) and protein (72%, 58%, and 52%; *p* < 0.01) expression levels of PAI-1 (Figure 2(A,B)) in the aorta for the HC + ASTX groups. No significant differences were found in the activity, mRNA expression, and protein expression of t-PA in the HC + ASTX groups (Figure 2(A–C)).

### Effects of ASTX on platelet aggregation, GMP140, TXB2 and 6-keto-PGF1α levels in hyperlipidemic rats

**Figure 5.**
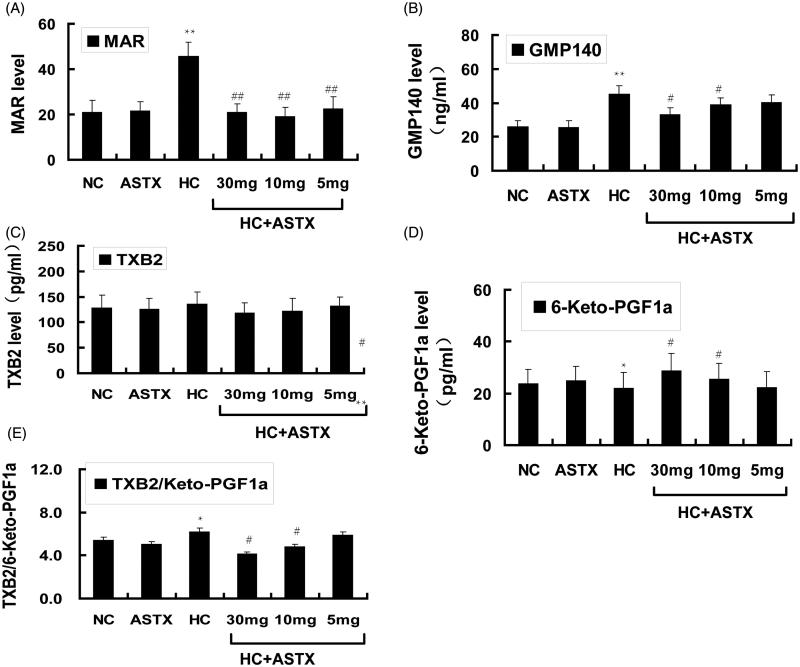
Effects of ASTX treatment on MAR, GMP140, TXB2 and 6-keto-PGF1α levels in hyperlipidemic rats. Experimental rats were fed a high-fat diet before treatment with solvent or ASTX. The platelet activity in the blood samples was determined. (A) MAR levels were determined with a coagulation analyzer. (B) Plasma GMP140 levels were determined by ELISA (ng/mL). (C) Plasma TXB2 levels were determined by ELISA (pg/mL). (D) Plasma 6-keto-PGF1α levels were determined by ELISA (pg/mL). (E) TXB2/6-keto-PGF1α ratio. Data are expressed as mean ± SEM (*n* = 12); *p < 0.05, **p < 0.01, compared with rats fed with a normal diet; #*p* < 0.05, ##*p* < 0.01, compared with rats fed with a high-fat diet.

Platelet activation often accompanies the release of reactive hyper function substances. Several substances are expressed in the surfaces of platelets or released into the bloodstream, we determined the changes in these substances. Compared with the NC group, treatment with ASTX alone did not significantly influence platelet function and the related active substances. The MAR (2.2-fold, *p* < 0.01; Figure 5(A)) and GMP-140 (1.7-fold, *p* < 0.01; Figure 5(B)) levels and TXB2/6-keto-PGF1α rates (Figure 5(E)) increased in hyperlipidemic rats. By contrast, the 6-keto-PGF1α levels (Figure 5(D)) mildly decreased, whereas the TXB2 levels (Figure 5(C)) mildly increased in hyperlipidemic rats. Compared with the HC group, ASTX treatment at 30, 10 and 5 mg/kg decreased the MAR levels (55%, 59% and 50%; *p* < 0.01; Figure 5(A)). ASTX was beneficial to the GMP-140 levels only when administered at 30 mg/kg (25%; *p* < 0.05; Figure 5(B)). ASTX was beneficial to the TXB2/6-keto-PGF1α rates (34 and 23%, *p* < 0.05; Figure 5(E)) and 6-keto-PGF1α levels (1.3- and 1.2-fold, *p* < 0.05; Figure 5(D)) but only when administered at doses of 30 and 10 mg/kg, respectively. The TXB2 levels were not significantly different between the HC + ASTX and HC groups.

## Discussion

Hyperlipidemic rats treated with ASTX in the present research showed anticoagulation and profibrinolytic activity, as well as decreased platelet aggregation. Our results were similar to those of previous reports (Bai et al. 2006) because haemostatic systems are involved in the coagulation system, fibrinolytic system and platelet aggregation. In the current work, we explored how ASTX affects these three aspects of haemostatic systems.

The normal lipid and lipoprotein levels provide a surface for the optimal activation of pro- and anticoagulant enzymatic complexes. In hyperlipidemia, the variation in lipid and lipoprotein levels can upset the balance between pro- and anticoagulant pathways, as well as provide a surface for the activation of pro-coagulation enzymatic complexes, namely, the activation of the surface increased factor Xa and factor Xa/Va-mediated factor VII (Kjalke et al. 2000; Olivieri et al. 2013). These clotting factors can activate the coagulation cascade. Our results showed shorter PT and APTT in hyperlipidemic rats than in normal rats.

However, ASTX can normalize the aforementioned changes. Our results showed that ASTX prolonged PT and APTT in hyperlipidemic rats. Two effects of ASTX involved the coagulation systems.

1. ASTX decreased the lipid and lipoprotein levels and restored the plasma lipid levels to near-normal levels (Arunkumar et al. 2012). Ryu et al. (2012) observed that the oral administration of the novel ASTX prodrug CDX-085 lowers the total cholesterol and aortic arch atherosclerosis in LDLR^(−/−)^ mice, as well as the triglyceride levels in ApoE^(−/−)^ mice. Consistent with their report, our experiments also showed that ASTX not only significantly reduced the TG, TC and LDL-C levels but also increased the HDL-C content and mildly affected the apoB100, apoE and apoAI levels in hyperlipidemic rats. The increased energy expenditure caused by ASTX (Ikeuchi et al. 2007) increased LDL uptake, upregulated carnitine palmitoyltransferase-II (Arunkumar et al. 2012) and increased fatty acid β-oxidation (Lee et al. 2003; Yang et al. 2011).
2. ASTX inhibits peroxyl radical-induced lipid peroxidation (Stahl & Sies 2012) and oxLDL production (Yuan et al. 2011). Our research showed that ASTX administration significantly decreased MDA levels but increased SOD and GPx activities in hyperlipidemic rats. The results also confirmed the stronger antioxidant function of ASTX. ASTX displayed non-enzymatic and enzymatic antioxidant activity. Non-enzymatic antioxidant activity depends on the special structure of ASTX. ASTX has keto and hydroxyl moieties, one on each of its two ionone rings; its transmembrane region is positioned in the phospholipid bilayer of cell membranes. ASTX exposes its hydrophilic ends in aqueous environments and removes ROS via a conjugated double-bond system of its hydrocarbon backbone (Riccioni et al. 2012). ASTX functions as an enzymatic antioxidant by increasing Nrf2 expression (Motohashi & Yamamoto 2004; Kaspar et al. 2009. ASTX can induce HO-1 expression by activating the ERK signalling pathway (Wang et al. 2010). By contrast, the ERK MAPK and JNK MAPK pathways activate Nrf2 via the transcription activating factor CBP (Huang et al. 2002). Thus, ASTX increases the Nrf2 expression-induced activation of the ERK signal pathway. Nrf2 can increase the expression of its downstream targets, such as SOD and GPx (Aleksunes & Manautou 2007; Li et al. 2009); therefore, SOD and GPx can remove ROS.

Hyperlipidemic rats exhibited increased plasma LP(a) levels, which inhibited t-PA activity and enhanced LP(a)-induced PAI-1 production in vascular endothelial cells (Ren et al. 1997). In our study, hyperlipidemic rats showed higher levels of serum FG, D-D and LP(a), as well as increased PAI-1 and t-PA activities. However, the elevation of PAI-1 activity was more significant. These results demonstrated the dysfunction of the fibrinolytic system in hyperlipidemic rats. However, the change in t-PA activity was inconsistent with previous reports (Ren et al. 1997). The reason for this difference remains unclear. We speculated that the observed PAI-1 and t-PA activity was caused by the injured vascular endothelium, which increased their release. ASTX could improve the fibrinolytic system.

Serebruany et al. (2010) reported that ASTX *in vitro* does not affect platelets, coagulation or fibrinolytic indices in either aspirin-naive or aspirin-treated subjects. By contrast, Chan et al. (2012) reported that ASTX intake at 0.05% significantly diminishes the activity of plasminogen activator inhibitor-1 and factor VII but enhances antithrombin-III and protein C activity in circulation. Our research supported the results of the latter study, such that ASTX decreased the serum FG, D-D and LP(a) levels. ASTX decreased PAI-1 activity and significantly downregulated the PAI-1 mRNA and protein expression levels in the aorta of hyperlipidemic rats. ASTX decreased PAI-1 activity by downregulating PAI-1 expression. Under several conditions, the lower PAI-1 activity often followed the higher t-PA activity. However, in our study ASTX only mildly increased t-PA activity, as well as upregulated the mRNA and protein expression levels. The results showed that ASTX mainly regulated fibrinolytic systems by significantly decreasing PAI-1 expression and activity while relatively increasing t-PA activity in hyperlipidemic rats. LP(a) is structurally homologous to t-PA and can compete with t-PA effects. ASTX also increased t-PA activity by decreasing the LP(a) levels.

Both t-PA and PAI-1 are mainly produced in the vascular endothelium. The altered expression of t-PA and PAI-1 was closely related to the vascular endothelium. In hyperlipidemic rats, lipid disorders initiated oxidative stress, in which reactive oxygen species (ROS) oxidized LDL, VLDL, and HDL *in vivo*. Specifically, LDL was oxidized to ox-LDL. ROS and ox-LDL impair endothelial cells and platelets to increase PAI-1 expression (Maruyama 1998). Endothelial dysfunction is commonly demonstrated by impaired endothelium-dependent vasorelaxation, such as the presence of NO in the vessel wall and blood serum (Jin et al. 2009). Impaired vascular endothelium alters eNOS metabolism and decreases the NO levels via a complex process. In hyperlipidemic rats, ROS exceeded the available antioxidant defence systems. NADPH oxidases were upregulated in the vascular wall and generated superoxide (O_2_^-^). O_2_^-•^ and NO rapidly recombined to form peroxynitrite (ONOO^−^), which could oxidize the essential cofactor of eNOS, (6*R*-)5,6,7,8-tetrahydrobiopterin (BH 4) into the trihydrobiopterin radical (BH 3) and 6,7-[8H]-H2-biopterin (BH 2). Consequently, oxygen reduction and O_2_ reduction by eNOS were uncoupled from NO formation, but O_2_ was produced. The functional NOS was converted into dysfunctional O_2_^-•^, thereby generating catalysts that contribute to vascular oxidative stress (Förstermann 2010). Our results also confirmed that hyperlipidemia increased eNOS activity but decreased the NO levels. The impaired vascular endothelium also decreased the secretion of PGI2 (Lee et al. 2003). The low serum NO and PGI2 levels may benefit haemostasis by vasoconstriction.

ASTX can protect the vascular endothelium. Sasaki et al. (2011) found that ASTX spontaneously protects hypertensive stroke-prone rats from vascular oxidative damage, hypertension and cerebral thrombosis. Riccioni et al. (2012) reported that ASTX can ameliorate endothelial inflammation and oxidative stress. Our research also showed that ASTX increased the NO and 6-keto-PGF1α (PGI2 stable metabolites) levels. ASTX increased the 6-keto-PGF1α level by restoring endothelial function. The regulation of NO levels by ASTX is complex. Our results showed that treatment with ASTX did not affect eNOS activity in hyperlipidemic rats. However, ASTX downregulated the mRNA and protein expression of eNOS in hyperlipidemic rats. These phenomena could be attributed to ASTX, which could protect the vascular endothelium and decrease the endothelial eNOS expression induced by various stimuli. ASTX could normalize the function of eNOS to produce NO by preventing eNOS ‘uncoupling’ because of two possible reasons: (1) ASTX inhibits the secretion of ROS or reactive nitrogen species (Lee et al. 2003; Lauver et al. 2008). (2) ASTX can react with peroxynitrite to form nitro derivatives (Hayakawa et al. 2008). ASTX can scavenge peroxynitrite and limit the oxidization of peroxynitrite to BH4. The BH4 levels increase, whereas the binding of BH4 and l-arginine is enhanced. The oxygen reduction and O_2_ reduction by eNOS are coupled from NO formation. Consequently, the serum NO levels are increased. The normal serum NO and PGI2 levels benefit from the antihemostatic effect via vasorelaxation.

Hyperlipidemia can alter platelet function. Lipid disorders change the lipid components of platelets. The lipid component variation in platelet membranes also lowers membrane mobility (Sener et al. 2005). A special LDL–oxLDL mechanism induces platelet adhesion, aggregation and secretion of granule contents (Levin & Egorihina 2013), such as P-140, TXA (2) and PAI-1 (Mahfouz & Kummerow 2000). Peroxynitrite causes endothelium apoptosis and platelet activity (Moncada et al. 1991). In our study, hyperlipidemic rats demonstrated increased levels of GMP-140 and TXB2 (TXA2 stable metabolite)/6-keto-PGF1α rates. These results showed that the platelets were activated. Simultaneously, the platelet function index and platelet MAR significantly increased. Thus, ASTX could restore the normal function of platelets. Lauver et al. (2008) also found that platelet aggregation and thrombus weights are inhibited by DDA in a dose-dependent manner. Our results showed that ASTX reduced the serum GMP-140 levels, TXB2/6-keto-PGF1α rates and platelet MAR. However, ASTX had almost no effect on the TXB2 levels. ASTX decreased the GMP-140 levels by stabilizing platelet membranes and inhibiting GMP-140 secretion and release. The effects of stabilizing platelet membranes rely on the normalization of lipid levels by ASTX. ASTX also inhibited platelet aggregation by elevating NO and 6-keto-PGF1α levels (Moncada et al. 1991).

Our study systematically investigated the effects of ASTX on blood clots. Results showed that ASTX could inhibit coagulation, increase fibrinolytic activity and reduce platelet aggregation in hyperlipidemic rats. This action was mainly attributed to ASTX, which decreased the serum lipid and lipoprotein levels, increased the amount of antioxidants, and protected the endothelial and platelet functions. This mechanism may be involved in the ASTX-influenced maintenance of the NO/ROS, t-PA/PAI-1, and TXA2/PGI2 balance. This work showed that ASTX did not directly change t-PA activity and expression to alter the t-PA/PAI-1 balance. ASTX mainly regulated the fibrinolytic system by significantly decreasing PAI-1 expression and activity while relatively increasing t-PA activity. For the NO/ROS balance, ASTX also did not change eNOS activity but increased NO by restoring ‘uncoupled’ eNOS. For the TXA2/PGI2 balance, ASTX mainly increased PGI2 expression but also produced a relative decrease in the TXB2/6-keto-PGF1α rates to inhibit platelet aggregation. A major limitation of the present study is that ASTX decreased oxidative stress. Therefore, we cannot rule out the possibility that ASTX inhibited coagulation, increased fibrinolytic activityand reduced platelet aggregation by decreasing oxidative stress levels.

In conclusion, we demonstrated that ASTX could inhibit coagulation, increase fibrinolytic activity and reduce platelet aggregation in hyperlipidemic rats. These results indicate that ASTX is an effective complementary and alternative antihaemostatic drug.
